# Breaking Through the Barrier: Nanoparticle-Driven MRI Strategies for Diagnosis and Therapy of Pancreatic Cancer

**DOI:** 10.1021/acsnanoscienceau.5c00116

**Published:** 2025-12-16

**Authors:** Alessandro Amaolo, Angelo Scarciglia, Marianna Sorrentino, Enza Di Gregorio, Giuseppe Ferrauto

**Affiliations:** 1 Molecular Imaging Center, Department of Molecular Biotechnology and Health Sciences, University of Turin, Via Nizza 52, Turin 10126, Italy; 2 Centro Alti Studi Difesa, Scuola Superiore Universitaria (CASD/SSU), Piazza delle Rovere 84, Rome 00165, Italy

**Keywords:** contrast agents, magnetic resonance imaging (MRI), nanoparticles (NP), pancreatic ductal adenocarcinoma (PDAC), stromal barriers, theranostics, tumor microenvironment (TME), tumor targeting

## Abstract

Pancreatic ductal adenocarcinoma (PDAC) remains a highly lethal cancer due to late diagnosis, limited biomarkers, and a dense stromal microenvironment that hinder imaging and therapy. Magnetic resonance imaging (MRI) offers high soft-tissue resolution but lacks molecular sensitivity and specificity when applied to PDAC. Advanced MRI methods such as diffusion-weighted imaging (DWI), hyperpolarized MRI, MR elastography (MRE), and dynamic contrast-enhanced MRI (DCE-MRI) have expanded the ability to characterize tumor microstructure, metabolism, stiffness, and perfusion, offering valuable functional insights. Nanoparticle-based contrast agents are emerging as promising tools to overcome these limitations by improving sensitivity, targeting tumor biomarkers, and enabling theranostic applications. This review explores the frontiers of nanoparticle-enhanced MRI in the context of PDAC. Recent studies show that superparamagnetic iron oxide nanoparticles (SPIONs) enhance T2-weighted MRI contrast and detect small PDAC lesions *in vivo.* Gold-based nanostructures complexed with gadolinium ions demonstrated relaxivity up to 7-fold higher than standard agents at 3T when compared to Gadovist, providing brighter tumor imaging. Liposomal formulations carrying gadolinium or collagenase improve tumor accumulation and stromal penetration, while multifunctional PLGA-based systems combine drug delivery with MRI tracking. Importantly, nanoparticle strategies also enable detection of high-risk precursors such as PanINs through targeting of early biomarkers, including MUC1, MUC4, and CLDN4. By bridging nanotechnology, molecular imaging, and cancer biology, this review presents a comprehensive perspective on the next-generation MRI tools that could redefine early detection and treatment monitoring in pancreatic cancer. Addressing challenges of stroma penetration, biodistribution, and safety will be critical for their translation into clinical practice.

## Graphical abstract

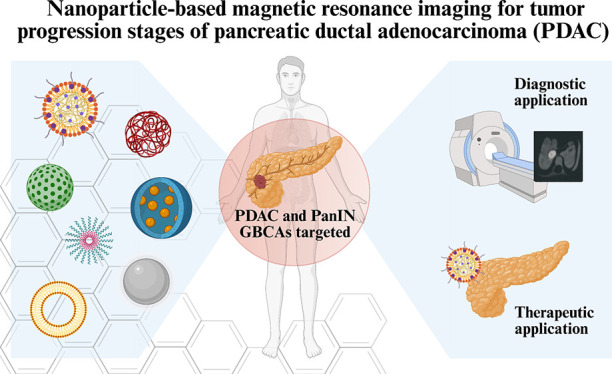

## 1 Introduction

Over 90% of malignant pancreatic tumor cases consist of pancreatic ductal adenocarcinoma (PDAC), making it by far the most frequent pancreatic malignancy.^1–3^ Despite numerous efforts, pancreatic cancer persists as one of the deadliest cancers worldwide as, according to the American Cancer Society, the 5-year survival rate is 13% across all stages combined, ranging from 44% for localized disease to 3% for distant metastatic disease. Pancreatic cancer represents the third leading cause of cancer death in the United States, causing over 50,000 deaths every year, and is projected to become the second leading cause of cancer death by 2040.^4–7^ Over the last 30 years, pancreatic cancer incidence has increased by about 1% per year, while mortality has risen more modestly from approximately 0.2 to 0.3% annually,^5^ whereas both the incidence and mortality of lung and colorectal cancers have declined.^4,5,8,9^ In Figure 1, pancreatic cancer incidence and mortality rates nearly overlap, indicating a mortality-to-incidence ratio approaching unity (Figure 2).

**Figure 1:**
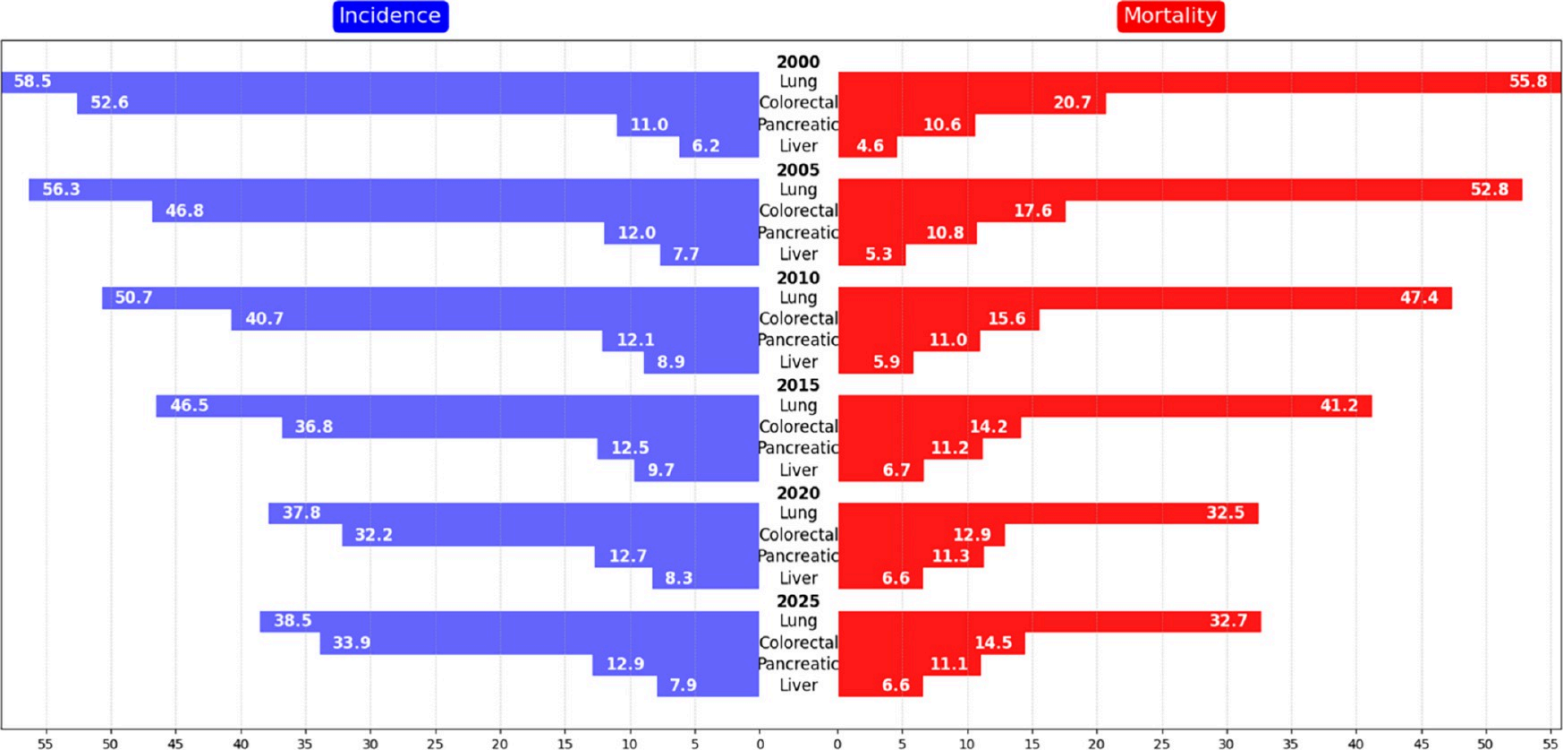
Age-standardized (World) per 100,000 individuals, in USA, reported as incidence and mortality, data from SEER,^10^ *estimated projection for 2025.

**Figure 2:**
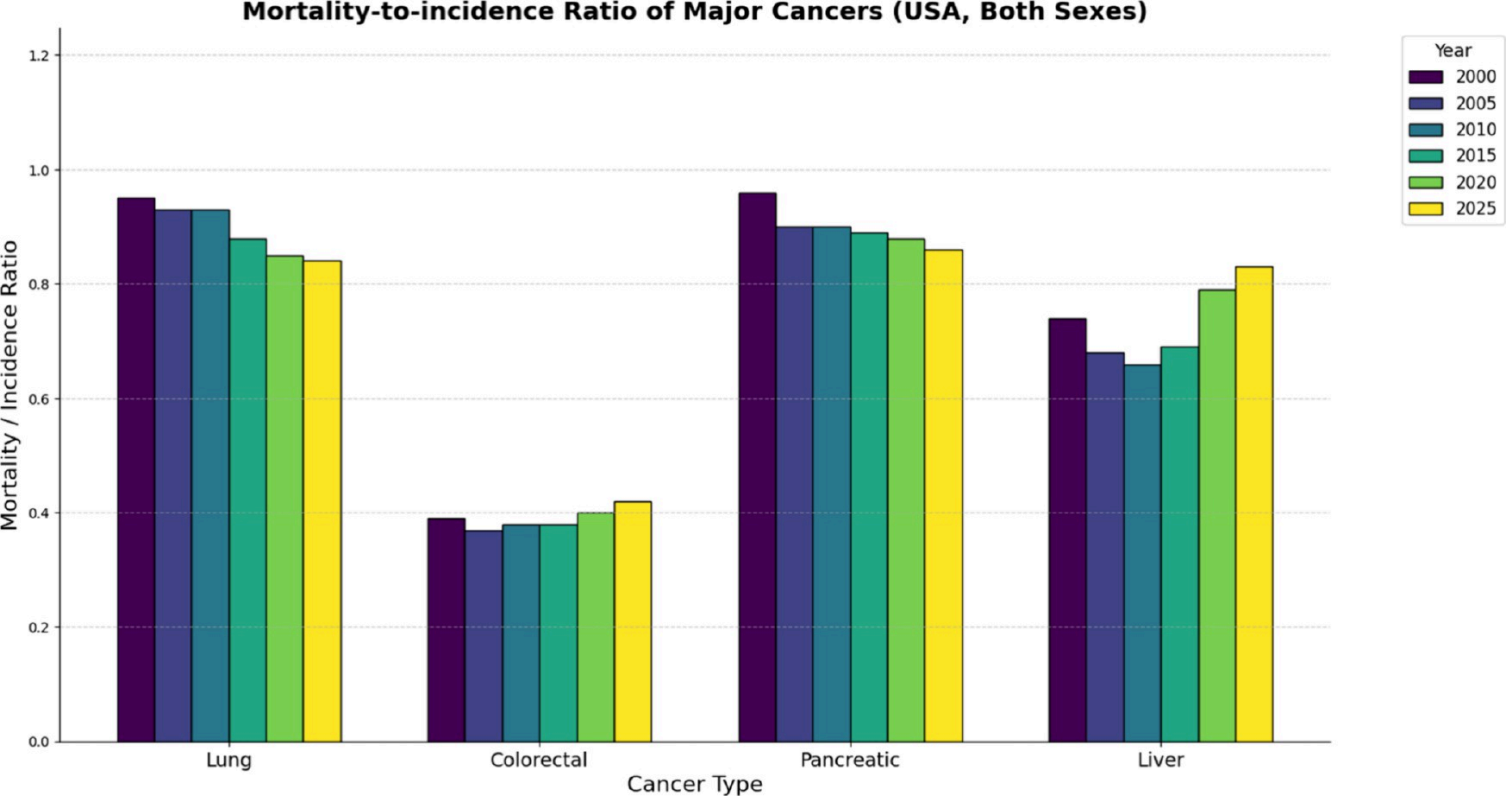
Data as mortality-to-incidence ratio on scale,^10^ *estimated projection for 2025.

This underscores the pressing need for enhanced treatment modalities. The current gold standard treatment options include surgical interventions, chemotherapy, and diverse forms of radiation therapy.^11^ Timely detection is paramount for effective intervention, highlighting the necessity of novel diagnostic and therapeutic approaches. The integration of nanotechnology with complementary disciplines, such as molecular biology and imaging technology, offers distinct capabilities, fostering innovation in diagnosis and treatment. Moreover, it facilitates personalized therapy and monitoring, accommodating individual patient variability, and optimizing treatment efficacy.

Compared to other imaging techniques, MRI provides superior spatial and temporal resolution, the possibility to image deep tissues and, among all, the use of nonionizing and noninvasive radiations.^12^ However, MR quantitative imaging of the pancreas remains challenging due to the small size of the pancreas and its sheltered location in the abdominal cavity.

Contrast-enhanced MRI (CE-MRI), employing Gd-based MRI contrast agents (GBCAs, Figure 3) can increase the capability to detect cancers, allowing a better discrimination of diseased tissues from healthy ones, owing to endogenous differences in longitudinal and/or transverse water proton relaxation times (*T*_1_ and *T*_2_, Figure 4).

**Figure 3:**
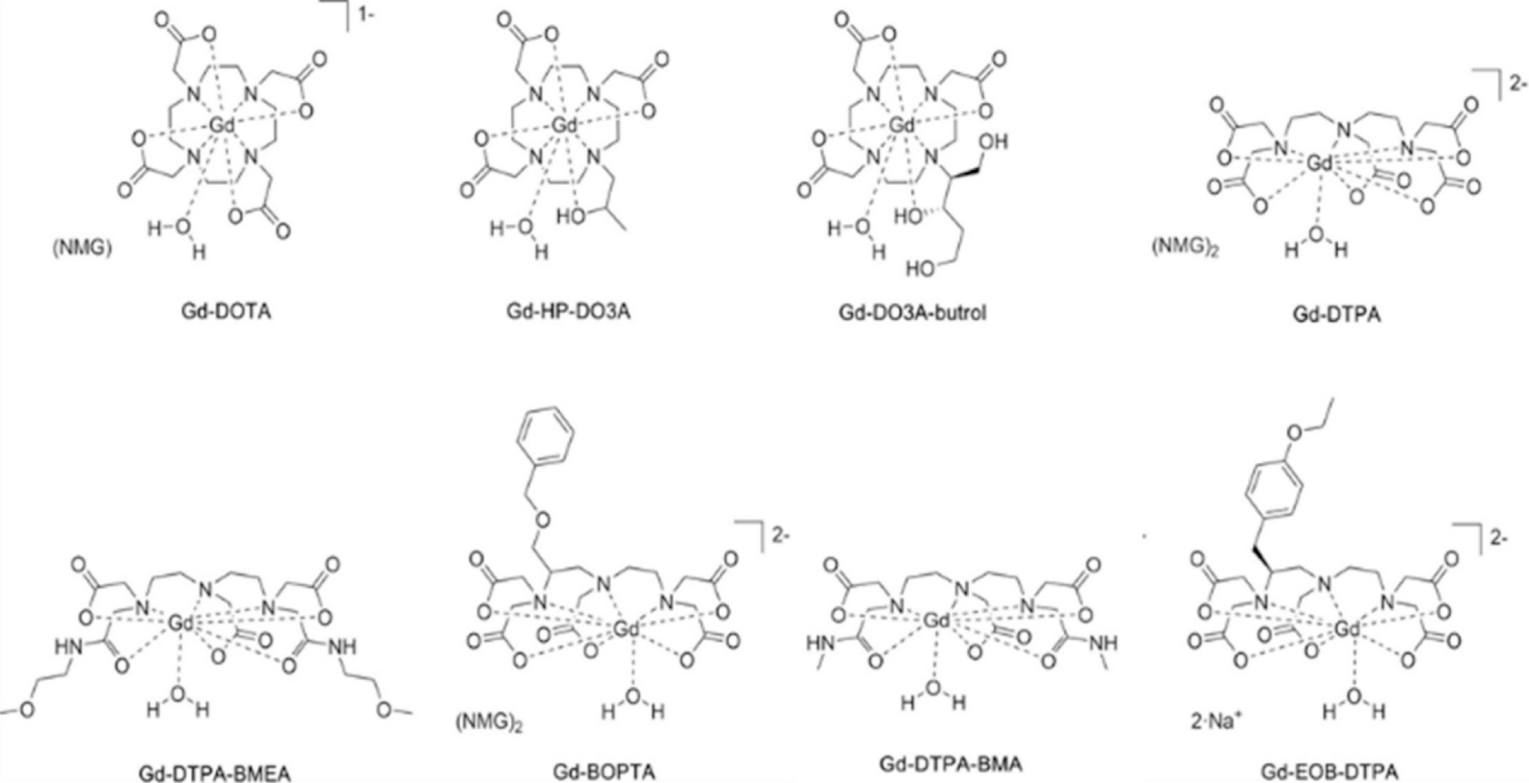
Commercially approved GBCA.

**Figure 4:**
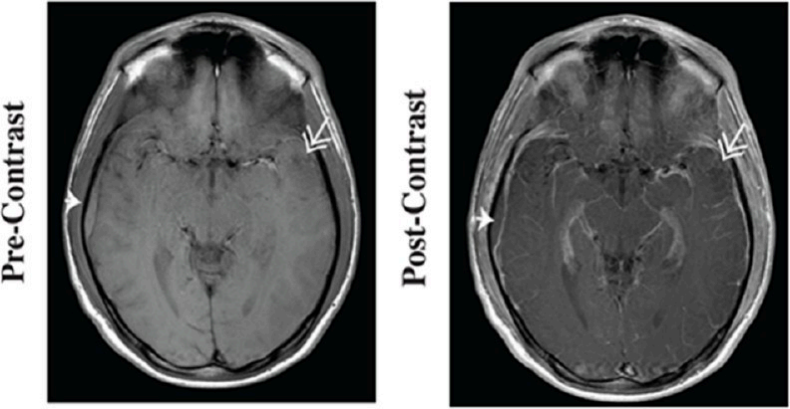
*T*_1w_ images pre- and post GBCA injection. Reprinted or adapted with permission under a Creative Commons CC BY 4.0 license from 14. Copyright 2020 PLOS ONE.^13^

The main problem related to GBCA CE-MRI is the low sensitivity of MRI, which requires the delivery of a large number of GBCAs to the PDAC region. Combining GBCAs and the unique properties of nanoparticles (NPs) can represent a unique opportunity to enhance MRI by increasing tumor local delivery, thus improving the image contrast at the site of interest. This Perspective highlights the current state of the art of MRI diagnosis and management techniques, specifically focusing on the potential of integrating nanotechnology with molecular imaging and challenges in using NP-based contrast agents for diagnosis.

While NP-based imaging agents have been extensively reviewed in the literature,^14–16^ most of these works address nanoplatform design and multimodal applications in oncology more broadly. Our review differs by focusing specifically on MRI applications in PDAC, a malignancy with unique biological barriers and an extremely poor prognosis. We emphasize the interaction between NPs and the dense desmoplastic stroma, highlighting how this microenvironment limits both passive and active targeting. Furthermore, we provide a detailed discussion of theranostic nanoplatforms in PDAC, linking imaging performance with drug delivery strategies and early lesion detection (PanINs). This targeted focus distinguishes our work from general reviews, positioning it as a PDAC-centered perspective that integrates nanotechnology, molecular imaging, and stromal biology.

## 2 Role of MRI in PDAC Diagnosis and Management

The clinical aims of diagnostic imaging in PDAC are to assess tumor stages and progression, determine primary tumor resectability, evaluate distant metastases, and monitor treatment response. The principal modalities for imaging the pancreas include computed tomography (CT), MRI, and endoscopic ultrasonography (EUS); however, their applications are limited to diagnosis and staging. MRI has gained significant value for early detection in high-risk cohorts, such as individuals with a family history of the disease or diabetics, and for differentiating precancerous pancreatic lesions.^17,18^ Its noninvasiveness and superior accuracy make it the preferred mode of diagnosis over the other techniques.^19^ A systemic review and meta-analysis on PDAC diagnosis showed that MRI has a sensitivity, specificity, and diagnostic accuracy of 93, 89, and 90%, respectively^19^ (Table 1). MR and MR cholangiopancreatography (MRCP) are superior to CT in assessing pancreatic structures and the biliary tree and duct. These fluid-rich structures produce an endogenous *T*_2_ contrast enhancement giving the MR image an hyperintense appearance against the surrounding nonfluid containing tissues.^20–22^ Moreover, MRI is often preferred for cystic lesion surveillance or when CT is contraindicated due to the cumulative deleterious effects of ionizing radiation and when resectability of the tumor is questioned.^23,24^

**Table 1:** Comparison between Imaging Techniques in Clinical Practice^26,27^

**imaging technique**	**sensitivity**	**specificity**	**pros**	**cons**
MRI	93% (95% CI = 88–96)	89% (95% CI = 82–94)	superior soft-tissue contrast, noninvasive, excellent for detecting liver metastasis	lower sensitivity for small tumors, can be less specific in distinguishing PDAC from pancreatitis
CT	90% (95% CI = 87–93)	87% (95% CI = 79–93)	widely available, fast, good for assessing calcifications and vascular involvement	involves ionizing radiation, lower soft-tissue contrast compared to MRI
EUS	91% (95% CI = 87–94)	86% (95% CI = 81–91)	high sensitivity for small tumors, allows for biopsy, excellent for locoregional staging	invasive, operator-dependent, limited field of view
PET	89% (95% CI = 85–93)	70% (95% CI = 54–84)	useful in detecting metastasis, especially in combination with CT or MRI	limited use for small tumors, expensive, involves radiation exposure

Worrisome early features of pancreatic malignancy are linked to abnormalities within the intraductal papillary mucinous neoplasms (IPMN), a macroscopic mucinous neoplasm that contributes to PDAC development.^25^

Key aspects to monitor during diagnosis include thickening of the main duct greater than 1 cm, mural node greater than 5 mm, cystic growth greater than 5 mm per year, and cyst greater than 4 cm in diameter.^28^ IPMNs are multicentric in 20–40% of cases, which emphasizes the importance of monitoring it to enhance diagnostic value.^29,30^ Solid lesions are well depicted by MRI and MRCP. Their ability to capture variable fluid signal intensity combined with the heterogeneous separation of the solid components give better accuracy for assessing local involvement of precancerous events and early detection.^26^ MRCP using 3D turbo spin echo sequences are preferred over the 2D ones, as they provide higher image quality and morphological detail of the side branches of the pancreatic duct.^31^ However, MRCP alone is limited to functional assessment of the ducts. Intravenous secretin (sMRCP) administration enhances ductal anatomy visibility stimulating secretion of a bicarbonate-rich fluid from and within the pancreatic ducts to delineate abnormalities.^32^ In a cohort study of 900 volunteers, MRCP detected pancreatic cysts in 27.7% of the cases, while sMRCP improved visualization and detection accuracy.^33^ A 17-study systematic review and meta-analysis found that the prevalence of incidental pancreatic cystic lesions is nearly 24.8% of MRCPs, suggesting its use in early-stage chronic pancreatitis.^34^

sMRCP lacks a standardized clinical protocol and rare cases of acute pancreatitis (AP) have been previously reported following procedure, although risk can be lowered by screening patients for a personal or family history of pancreatitis.^35^

### 2.1 Advanced MRI Techniques for PDAC

#### Diffusion-Weighted Imaging (DWI)

DWI-MR maps water diffusion cell membranes and macromolecules, capturing alterations in the tissue microenvironment^36^ (Figure 5). It quantifies water diffusivity, described by the apparent diffusion coefficient (ADC), as well as microcirculation of blood flow.^37^ DWI can help distinguish between benign and malignant pancreatic lesions.^38^ This noninvasive lesion characterization can reduce the need for confirmatory biopsies of the pancreatic tissue. Typically, ADC values in PDAC are lower compared with normal pancreatic tissue. However, ADC quantification often fails to differentiate the different solid pancreatic neoplasms due to overlapping values. As reported by Yao et al.,^39^ respiratory-triggered DWI at 3T elucidated histopathological patterns, with statistical differences in ADC values among PDAC, solid pseudopapillary tumors, and neuroendocrine tumors (PanNETs). Intravoxel incoherent motion (IVIM) is a DWI-derived technique that can separate the diffusion and perfusion components of water molecules in tissues. IVIM parameters, such as the perfusion fraction (*f*), have shown potential for better characterization, with lower *f* values in PDACs compared to normal pancreas, chronic pancreatitis, and PanNETs.^40,41^

**Figure 5:**
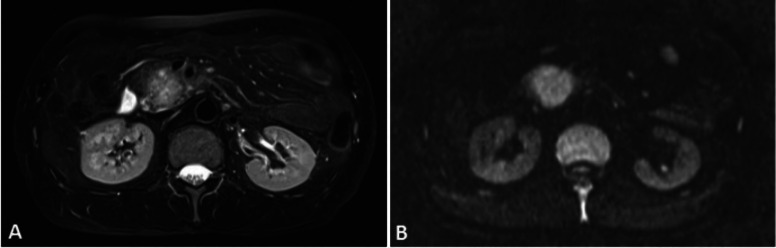
Representative pancreatic *T*_2‑weighted_ image (A) and DWI image (B). Reprinted or adapted with permission under a Creative Commons CC BY 4.0 license from ref 42. Copyright Oncotarget 2017 Impact Journals.^42^

Overall, DWI shows promise in distinguishing between benign and malignant lesions, and its ADC quantitative analysis can be challenging, although novel supporting uses such as IVIM parameters hold potential in diagnosis of PDAC.

#### Hyperpolarized MRI

Hyperpolarized MRI is a technique capable of identifying metabolic abnormalities in the pancreas that foretell preneoplasia. It uses hyperpolarized agents such as ^13^C pyruvate to detect and monitor progression of lesions projected toward PDAC^43,44^ (Figure 6). Other experimental models have proved the feasibility of hyperpolarized MRI in differentiating exocrine pancreas, pancreatitis, and pancreatic cancer tissues by assessing the enzymatic conversion of pyruvate-to-lactate and pyruvate-to-alanine ratios, which are both linked to disease progression and treatment response.^43^ A pilot clinical study showed successful characterizations and differentiation of heterogeneous and hypoxic pancreatic tumors upon injection of enriched ^13^C pyruvate via [1-^13^C] lactate and [1-^13^C] alanine production.^45^ Hyperpolarized holds promise in early detection and monitoring of PDAC progression by quantifying metabolites production ratios; however, further clinical studies are needed to establish its efficacy for diagnostic applications.

**Figure 6:**
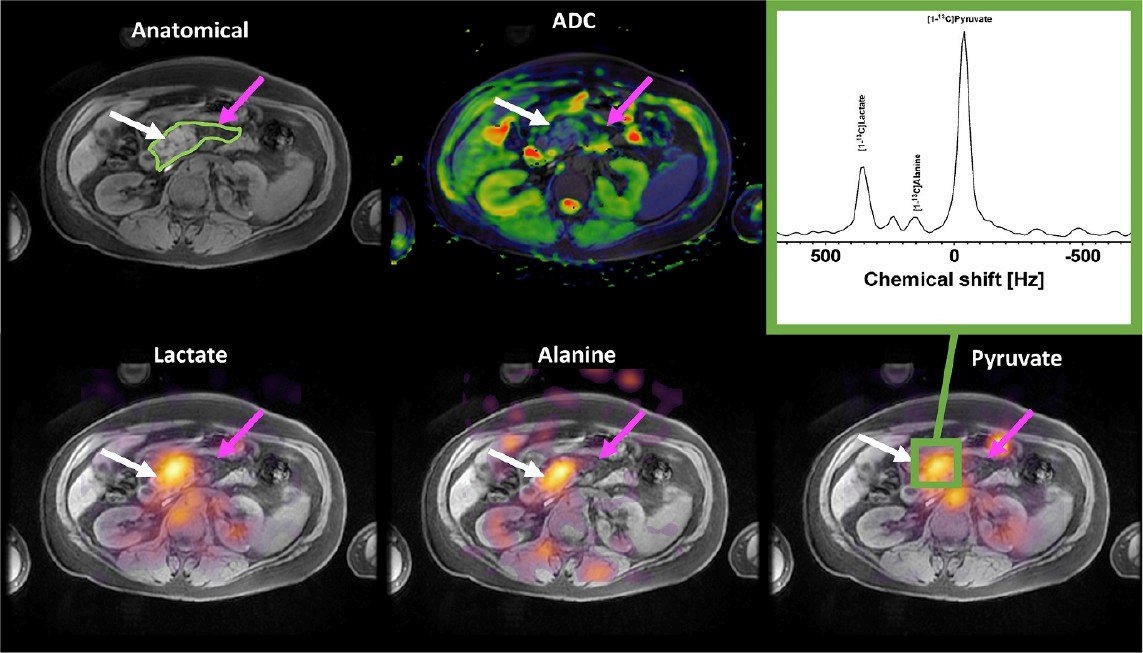
Top row: anatomical axial slice showing the pancreatic tumor. Bottom row: high [1-^13^C] pyruvate, [1-^13^C] lactate, and [1-^13^C] alanine signals were observed in the pancreatic tumor. Reprinted or adapted with permission under a Creative Commons CC BY 4.0 license from ref 45. Copyright 2020 John Wiley & Sons.^45^

#### MR Elastography (MRE)

MR elastography (MRE) is an emerging technique capable of detecting fibrosis’ stiffness and fluidity. Traditionally, stiffness is assessed by palpation, and fluidity is a relatively new indicator for tumor characterizations. MRE uses multi low-frequency vibrations to create a visual map to test tissue’s elasticity^46^ (Figure 7). MRE has proven to be promising in differentiating PDAC from pancreatitis with high accuracy. Zhu et al.^47^ compared stiffness measurements in PDAC and autoimmune pancreatitis (AP). Both stiffness and fluidity allowed higher detection rates and distinguishing among the two conditions.

**Figure 7:**
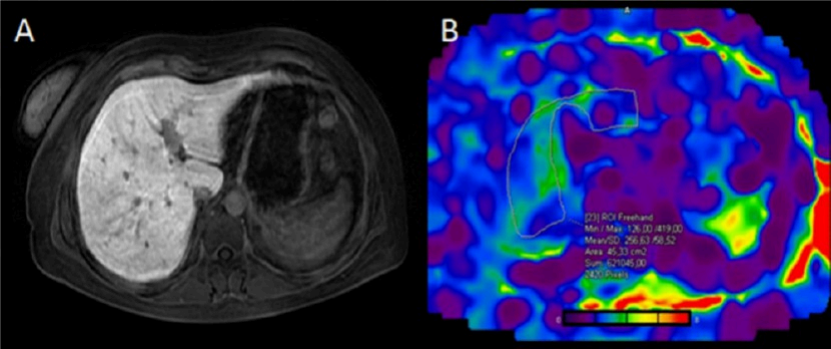
(A) MR image of the abdomen compared to the stiffness map (B) obtained with MRE. Reprinted or adapted with permission under a Creative Commons CC BY 4.0 license from ref 48. Copyright 2022 Springer Nature/Insights into Imaging.^48^

##### Dynamic Contrast-Enhanced MRI (DCE-MRI)

Dynamic contrast-enhanced MRI (DCE-MRI) is a valuable technique to assess tissue perfusion over time in the pancreas (Figure 8).

**Figure 8:**
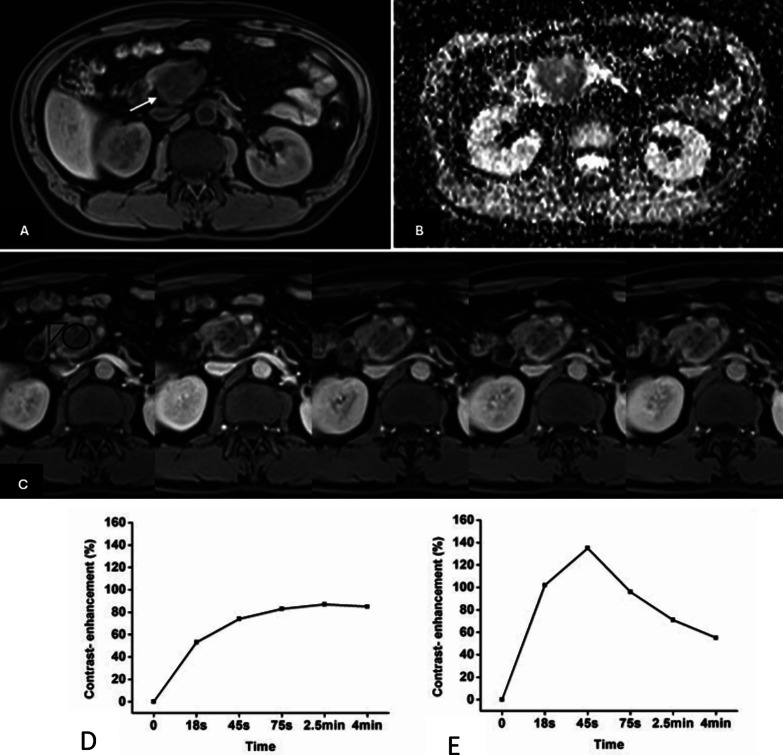
Representative pancreatic *T*1w image (A), ADC map (B), DCE-MR images (C), and (D, E) TIC profiles. The ROIs of mass and non-mass adjacent parenchyma (NAP) indicated with black circle and black triangle. Pancreatic mass demonstrates type IV b TIC with a slow increasing enhancement pattern followed by a plateau, while NAP demonstrates type II aTIC, which shows a rapidly increasing and then gradually decreasing enhancement pattern. DWI shows that pancreatic mass is clearly seen as hyperintense with a well-defined margin. Reprinted or adapted with permission under a Creative Commons CC BY 4.0 license from 43. Copyright 2017 Impact Journals.^42^

Applied even when there are no apparent morphological conditions, this imaging practice may be useful in high-risk PDAC patients. Granata et al.^49^ showed its greater sensitivity compared to IVIM-DWI and other imaging techniques, although its accuracy remains unclear since the factors that contribute to the analysis such as tumor hypoxia, angiogenesis and metastasis are still studied as markers of degree of fibrosis and vascularization.^50^

Advanced MRI techniques (Figure 9) are increasingly integrated into PDAC evaluation, offering functional and physiological insights and complementing traditional imaging. These approaches provide a deeper understanding of tumor biology, setting the stage for more detailed explorations.

**Figure 9:**
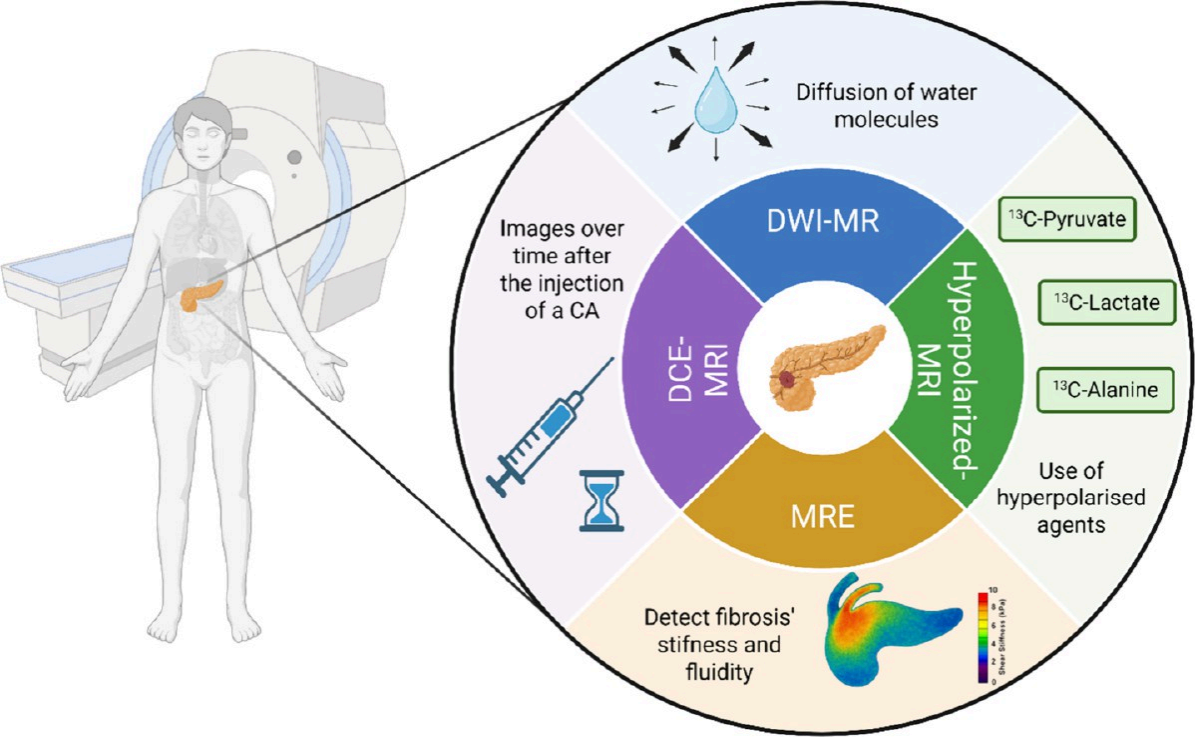
Applications of various MRI techniques in the detection, characterization, and staging of pancreatic ductal adenocarcinoma (PDAC).

This review focuses on the use of NP-based imaging techniques for advancing imaging in the early detection of PDAC, enabling an even earlier diagnosis than traditional imaging methods. Molecular imaging methods aim to deliver targeted probes and NP-based contrast agents to tumor-specific epitopes. Such candidates under investigation include tumor epithelial cells, plectin-1, receptor tyrosine kinase axl, bombesin receptors, and MUC4.^51,52^

The introduction of nanotechnology in molecular imaging offers a noninvasive method to enhance the bioavailability of therapeutic or imaging agents at the site of interest, improving contrast between hypovascular tumors and normal parenchyma during imaging.^53^ Multifunctional NP systems are also available to amplify signals and increase tumor uptake, allowing for better imaging sensitivity in MRI applications. There are challenges that need to be addressed when using NP imaging agents such as time-dependent accumulation and subsequent organ-specific biodistribution in clinical applications.^53^ Performance optimization will be essential in ensuring the clinical success of future trials and NP formulations.

## 3 NP-Based Contrast Agents for MRI in PDAC Imaging

NPs possess intrinsic and unique features in optical and magnetic physiochemical properties. Their high surface-area-to-volume ratio also allows for surface functionalization with different molecules. This enhances MR contrast enhancement, increases biodistribution, boosts detection sensitivity, spatial and temporal resolution, and supports multifunctionality and multimodal imaging capacity^54^ (Table 2).

**Table 2:**
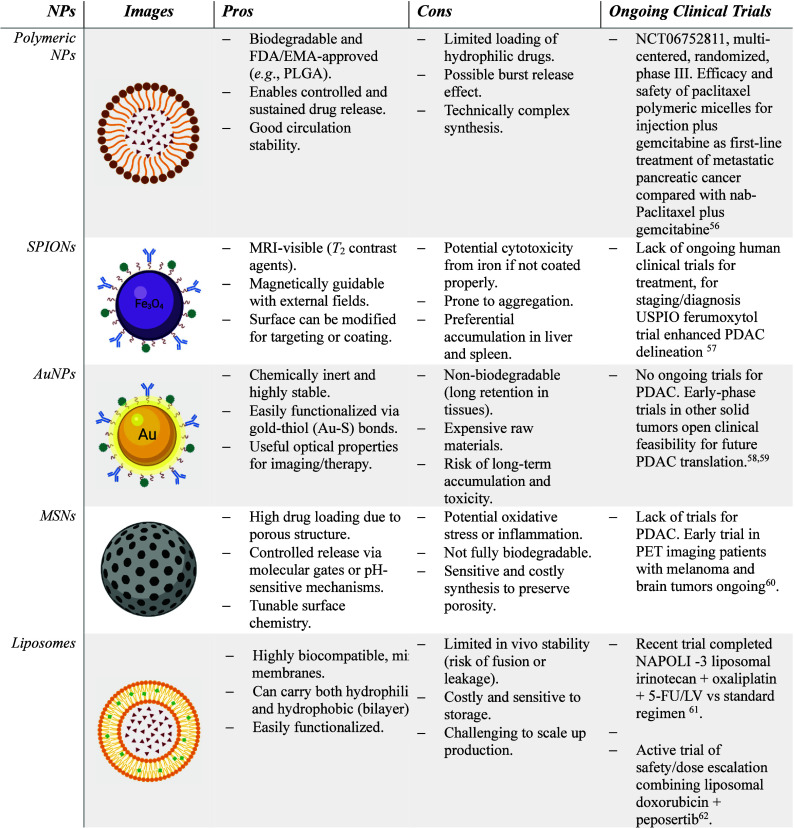
Summary Table of Nanoparticles for Targeted Imaging^57–62^

Traditional imaging agents alone, such as chelated gadolinium, lack the dual modality option that NPs can offer. Moreover, since they undergo rapid clearance throughout the systemic circulation, these small molecules have short *half-life*, restricting long-term follow-up. Various NP imaging probes based on polymeric and inorganic metals, iron oxide and carbon oxide nanoparticles, and liposomes have been evaluated for MRI in PDAC diagnosis.

### 3.1 Types of Nanoparticles for MRI in PDAC

MRI contrast relies on several complementary modalities, each probing different aspects of tissue biology. *T*_1_-weighted imaging provides high-resolution anatomical detail and can be enhanced by agents such as gadolinium, while *T*_2_-weighted imaging is particularly sensitive to water content and iron-based agents. DWI captures water mobility to reflect tissue cellularity; DCE-MRI measures vascular perfusion; MRE maps tissue stiffness; and hyperpolarized MRI detects real-time metabolic activity. In PDAC, each of these techniques provides valuable diagnostic information but is often limited by low sensitivity, rapid clearance of small-molecule agents, or poor lesion specificity.

NPs offer a way to overcome these limitations. Their tunable size, surface chemistry, and capacity to carry multiple payloads enable stronger *T*_1_ or *T*_2_ relaxation effects, targeted accumulation in tumor tissue, and integration with functional modalities such as DWI, DCE, and hyperpolarized MRI through the delivery of metabolic- or stromal-modifying probes. Moreover, many nanosystems combine imaging with therapeutic functions, making them suitable for theranostic applications. Against this background, a variety of NP platforms—including polymeric carriers, superparamagnetic iron oxide NPs (SPIONs), gold-based nanostructures, mesoporous silica nanoparticles (MSNs), and liposomes—have been explored for PDAC imaging.

#### Polymeric NPs

Polymeric NPs are the most versatile carriers in nanomedicine, widely studied for imaging and drug delivery in PDAC. They are typically made from biocompatible and biodegradable polymers such as poly­(lactic-*co*-glycolic acid) (PLGA) and polyethylene glycol (PEG). PEGylation increases the circulation time by reducing renal clearance and immune uptake, while PLGA provides structural stability and controlled degradation. These polymers can be loaded with targeting ligands such as gadolinium chelates, converting them into effective MRI probes.^53–55^ In a study, PLGA NPs encapsulating fluorescent iron oxide particles conjugated with HER2 antibodies enhanced *T*_2_-weighted contrast and simultaneously delivered gemcitabine, achieving tumor regression in subcutaneous MiaPaca2 xenografts.^65^ Such examples illustrate how polymeric NPs can combine drug delivery and imaging, with circulation half-lives extended to several hours and high specificity due to ligand functionalization.

#### Superparamagnetic Iron Oxide Nanoparticles (SPIONs)

SPIONs are well-known for their strong magnetic responsiveness, making them excellent *T*_2_-weighted MRI contrast agents. Depending on their size and surface chemistry, they can also be engineered to act as *T*_1_ enhancers. Functionalization with aptamers, peptides, or antibodies enables the active targeting of PDAC cells, overcoming the limitations of passive accumulation in the dense stroma. In vitro, SPION–PLGA composites showed concentration-dependent *T*_2_ signal darkening in MiaPaca2 and Panc1 cells.^66^ Antibody-conjugated SPIONs further improved imaging, with a transverse relaxivity of 104 mM^–1^ s^–1^ and in vivo *T*_2_ signal decreases of up to 30% at 24 h postinjection in PDAC xenografts.^67^ These results confirm SPIONs as powerful diagnostic and theranostic tools, although their rapid sequestration in the liver and spleen remains a translational barrier.

#### Gold NPs (AuNPs)

AuNPs are chemically inert, stable, and easily functionalized via gold–thiol chemistry. Although nonmagnetic, they can be hybridized with gadolinium or iron oxide to impart MRI contrast. Their optical properties also make them suitable for multimodal imaging, including CT and PA. Zhao et al.^68^ reported PEGylated Au nanorods coated with mesoporous silica and a gadolinium carbonate shell (AuNR–SiO_2_–Gd), which reached an *r*_1_ relaxivity of 46.40 mM^–1^ s^–1^ at 3T compared to 6.36 mM^–1^ s^–1^ for Gadovist.^56^ *In vivo*, these nanorods increased tumor-to-liver signal ratios from 1.75 to 6.10 over 48 h, enabling brighter and sustained tumor imaging in PDAC mouse models. Despite excellent performance, concerns remain about nonbiodegradability and potential long-term accumulation.

#### Mesoporous Silica NPs (MSNs)

MSNs have a highly porous architecture that allows high loading of drugs and contrast agents as well as controlled release triggered by pH, enzymes, or temperature. Functionalization with targeting ligands such as cyclic RGD (cRGD) peptides improves the specificity toward integrins overexpressed in PDAC vasculature. Li et al.^69^ demonstrated that Gd^3+^-doped MSNs functionalized with cRGD significantly enhanced tumor imaging in PDAC models, increasing tumor-to-background contrast compared to nontargeted MSNs.^69^ Their tunability makes them valuable theranostic carriers, although incomplete biodegradability and the potential induction of oxidative stress are challenges for long-term use.

#### Liposomes

Liposomes are lipid bilayer vesicles that can encapsulate both hydrophilic and hydrophobic contrast agents. They prolong circulation and can be modified for targeted delivery. In PDAC xenograft and patient-derived xenograft (PDX) mice, gadolinium-loaded liposomes achieved tumor accumulation of ∼1% of the injected dose, compared to rapid urinary clearance of free Gd.^70^ Collagenase-loaded liposomes improved stromal penetration,^71^ while solid magnetoliposomes combined with sonic hedgehog inhibitors tripled tumor deposition and *T*_2_ contrast after 24 h relative to controls.^72^ Clinically, liposomal irinotecan (Onivyde) is already approved for PDAC chemotherapy, underscoring the translational feasibility of this platform.^73^ The main limitations remain limited stability *in vivo* and manufacturing complexity.

#### Comparative Overview

Each NP type presents distinct strengths and weaknesses. Polymeric NPs offer biocompatibility and controlled degradation but may suffer from a low drug/contrast payload. SPIONs provide high *T*_2_ sensitivity and theranostic versatility but accumulate in liver and spleen. AuNPs exhibit unmatched multimodality and strong relaxivity but face nonbiodegradability concerns. MSNs enable high drug loading and functional versatility but raise safety issues related to oxidative stress and incomplete clearance. Liposomes are clinically advanced and biocompatible, although their tumor uptake is often modest. Overall, no single NP class solves all PDAC’s imaging challenges; instead, rational design and combination strategies are needed to maximize diagnostic sensitivity and therapeutic impact.

### 3.2 NP’s Targeting Strategies for Imaging PDAC

#### 3.2.1 Passive Targeting

Passive targeting relies on the enhanced permeability and retention (EPR) effect. First proposed by Maeda et al. in 1986,^74^ the EPR effect describes how nanoparticles of certain sizes preferentially accumulate in tumor tissues due to leaky vasculature and impaired lymphatic drainage and despite being considered a fundamental mechanism in preclinical models, its clinical relevance in human tumors, particularly in PDAC, remains subject to debate.^75^ The rapid growth of tumor cells compared to normal tissues leaves gaps between endothelial cells in the tumor vasculature, compromising lymphatic drainage and thus allowing extravasation of NPs. In practice, the efficiency of the EPR effect in solid tumors such as PDAC is limited. The stroma and high interstitial fluid pressure reduce vascular permeability, confining NPs to perivascular regions and preventing homogeneous penetration.^63^ EPR is highly variable between patients and tumor types, leading to inconsistent outcomes and making passive accumulation alone unreliable for imaging or therapy, with evidence suggesting that the effect is over-represented in preclinical xenograft models compared to clinical tumors.^76,77^ The true challenge of targeting PDAC stems from penetrating its dense stromal matrix, which impedes NP delivery but also increases interstitial fluid pressure,^64^ limiting intratumoral accumulation and therapeutic efficacy. To overcome these limitations, strategies to modify the TME, such as using matrix-degrading enzymes or agents that normalize tumor vasculature, have been developed.

#### 3.2.2 Active Targeting

Active targeting has emerged as an interesting strategy to improve the efficiency in the delivery of nanoparticles in PDAC. Unlike passive targeting, which depends on the tumor physiological properties, with the active targeting, it is possible to leverage specific molecular targets, undoubtedly increasing the uptake of NPs. Attaching ligands to their surface to bind to specific overexpressed receptors allows for enhanced imaging or treatment (Figure 10). With the variety of choice of cancer’s vulnerabilities, diverse targets for active targeting must be investigated to develop novel NPs involved in targeting PDAC for imaging and therapeutic purposes. Despite this potential, active targeting is still constrained by several factors. NPs must first reach the poorly perfused tumor site before ligands can bind, meaning that stromal barriers and low vascularization in PDAC strongly limit the delivery. Receptor expression is often heterogeneous and dynamic, reducing specificity over time. In addition, steric hindrance from the extracellular matrix and immune clearance of modified nanoparticles can impair binding efficiency.^78,79^ These limitations make active targeting an important complement, but not a complete solution, to the delivery problem in solid tumors.

**Figure 10:**
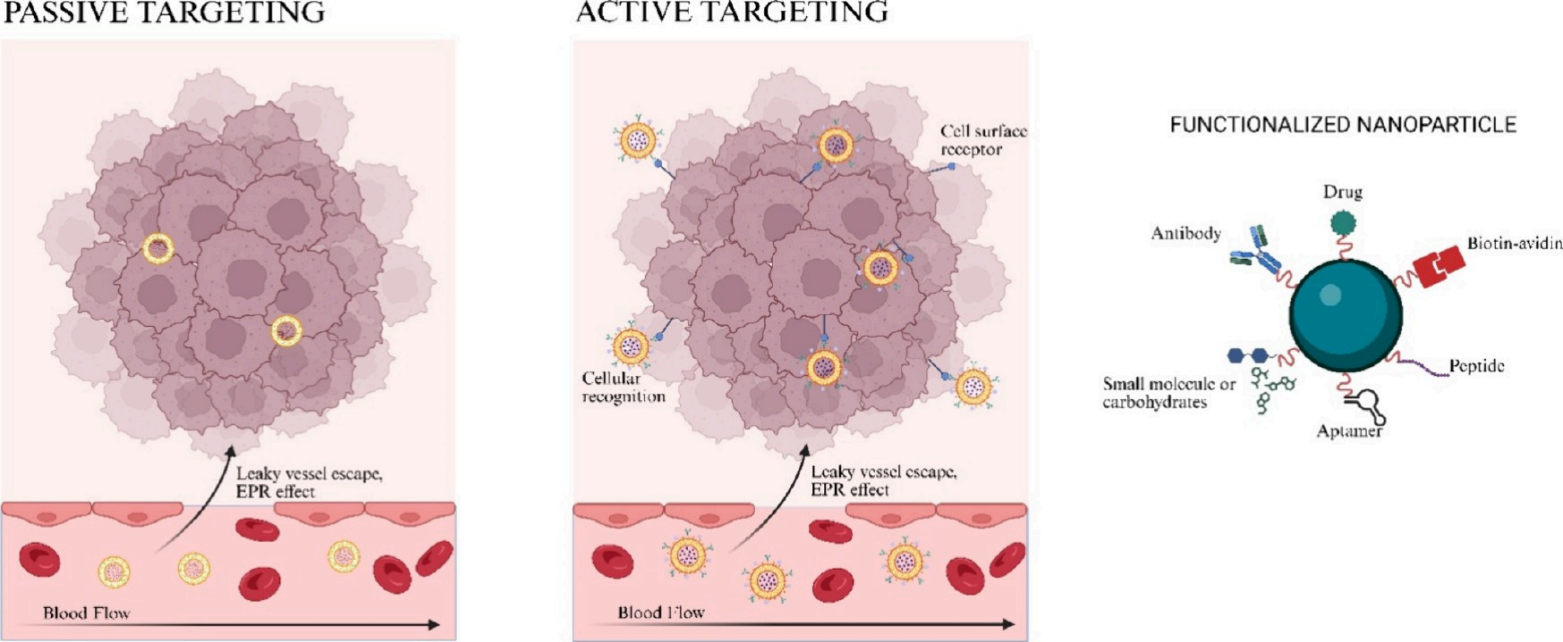
Active and passive targeting of NPs to tumor sites. Strategies of functionalization are highlighted.

##### Receptor-Mediated Uptake

Tissue plasminogen activator (tPA)-functionalized polymeric NPs showed high binding efficacy to galectins, overexpressed in PDAC cells in MR imaging^80^ (Figure 11). The incorporation of protease-activated outer PEG shell helps monocyte phagocytic system (MPS) evasion, enhancing their penetration, and thus imaging properties, through the TME in PDAC cells.^80^ *In vivo* application of these NPs showed enhanced tumor deposition and imaging sensitivity in both subcutaneous and orthotopic pancreatic tumor mouse models allowing for visualization of small PDAC tumors (<0.5 cm^3^).^80,81^

**Figure 11:**
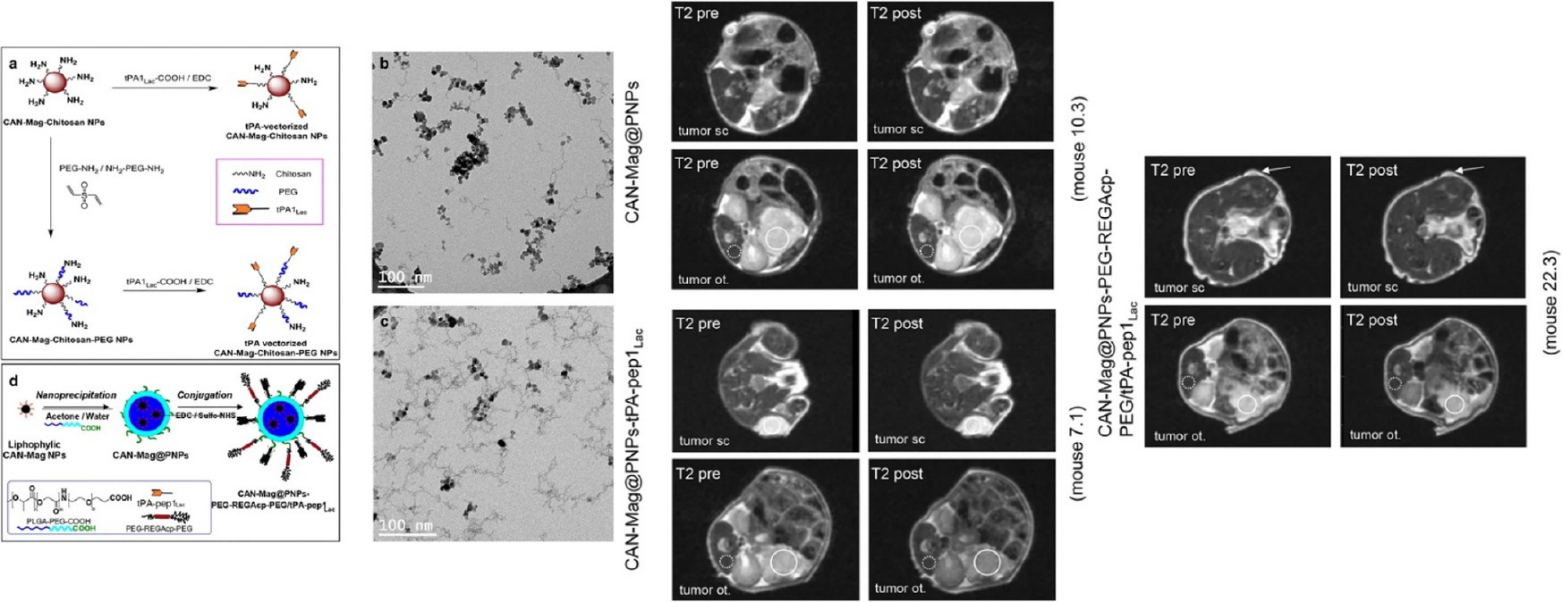
(a–d) Nanoparticle synthesis: a synthetic route to tPA decoration of both CAN-Mag-Chitosan and CAN-Mag-Chitosan-PEG NPs. TEM microphotographs of the NPs. (d) Final nanosystem CAN-Mag@PNPs-PEG-REGAcp-PEG/tPA-pep1_Lac_. MRI: a minor portion of the injected NPs reaches the tumor. T2-weighted images of a single slice before (*left*) and 5 min after injection (*right*) of CAN-Mag@PNPs; CAN-Mag@PNPs-tPA-pep1Lac; and CAN-Mag@PNPs-PEG-REGAcp-PEG/tPA-pep1Lac NPs injection. A signal drop (*darker area*) can be seen after NP application. Reprinted or adapted with permission under a Creative Commons CC BY 4.0 license from ref 80. Copyright 2016 Springer Nature.^80^

SPIONs embedded in a PLGA matrix enhance *T*_2_ MRI contrast.^82^ A concentration-dependent darkening effect on the *T*_2‑weighted_ images was observed. *In vitro* studies showed high cellular uptake by human pancreatic cancer cell lines, namely, MiaPaca2 and Panc1, particularly when ligated to specific tumor-targeting aptamers.^82^

Promising *in vivo* results further support the efficacy of polymeric-based nanocomposites. In a multifunctional study, PLGA NPs encapsulating gemcitabine and fluorescent iron oxide (FIO) particles, conjugated with human epidermal growth factor receptor 2 (HEGFR-2) antibodies, showed enhanced contrast MRI and tumor regression in subcutaneous MiaPaca2 xenografts in SCID mice.^83^

Overall, the advantages of using polymer-based NPs, particularly those encapsulating superparamagnetic iron oxide nanoparticles (SPIONs), show significant promise as contrast agents for MRI diagnostic applications in PDAC.

##### Multimodal Antibody-Conjugated NPs

Compared with the polymeric ones, inorganic NPs have greater tunability in terms of morphology, size, and surface chemistry. Their electrochemical and magnetic properties can be exploited for diagnostic imaging applications, such as MRI. AuNPs have gained attention as MRI contrast agents due to their inert and versatile functionalization. Their hydrophilicity contributes to their suitability for biomedical applications. Zhao et al.^68^ demonstrated that PEGylated Au­(III) nanorods encapsulated with a mesoporous silica matrix and gadolinium oxide carbonate shell (AuNR-SiO_2_-Gd) provide a trimodal contrast agent effective in MRI, CT, and photoacoustic in PDAC imaging. The *in vitro* results exhibited superior contrast in MRI when compared to conventional and approved agents like Gadovist. Specifically, Au-NR-SiO_2_-Gd NPs exhibited a longitudinal relaxation rate (*R*_1_) of 46.40 mM^–1^ s^–1^ whereas Gadovist only has 6.36 mM^–1^ s^–1^ at a magnetic field of 3T.^68^ The high relaxivity enhances MR signal intensity, making tissues “brighter” and more distinguishable. The *in vivo* application of this nanocomplex in Kras and p53 genetically engineered mouse models have further confirmed its efficacy. Although poorly distributed, NPs within the tumor appeared relatively brighter due to the accumulation of gadolinium in both *T*_1_-GRE and *T*_2_-FSE images consistent with the *in vitro* findings.^68^ However, from pre- to postinjection, tumors showed a signal-to-noise ratio (SNR) increase only in *T*_2_-FSE. The positive MR contrast in PDAC tumors at *T*_2_-weighted MRI, calculated as the ratio of SNRs between the tumor and liver, increased from 1.75 before injection to 5.43 at 24 h and to 6.10 at 48 h, highlighting the potential of AuNR-SiO_2_-Gd NPs in providing clear and sustained imaging of PDAC.^68^ Iron-based superparamagnetic NPs have been widely applied in MRI as *T*_2_ contrast agents.^84^ Conjugation with specific ligands, such as antibodies, peptides, and nucleotides, can result in vectorized multifunctional nanocomplexes.^85^ Pyruvate dehydrogenase 1 is a glycolytic enzyme upregulated at the mRNA/protein level in PDAC cell lines with an underlying influence in development, invasion, metastasis, and tumor chemoresistance.^86^ ENO1 also serves as a plasminogen receptor on the PDAC cell membrane, unlocking the possibility for molecular imaging with SPIONs.^86^ The assembly of SPIONs with the ENO1 antibody facilitates binding and internalization in CFPAC-1 and MiaPaca2 PDAC cell lines.^84^ Incubation of cells with ENO1-SPIO reduced *T*_2_ and *T*_2_* values, darkening the area of interest helping distinguishing tumor from pancreas parenchyma^84^ (Figure 12). The same results were obtained in *in vivo* PDAC xenograft nude BALB/c mouse models. Intravenous administration of targeted SPIONs followed the *in vitro* results as the *T*_2_ intensity at 1.5 T magnetic fields decreased postinjection with peak enhancement at 24 h.^84^

**Figure 12:**
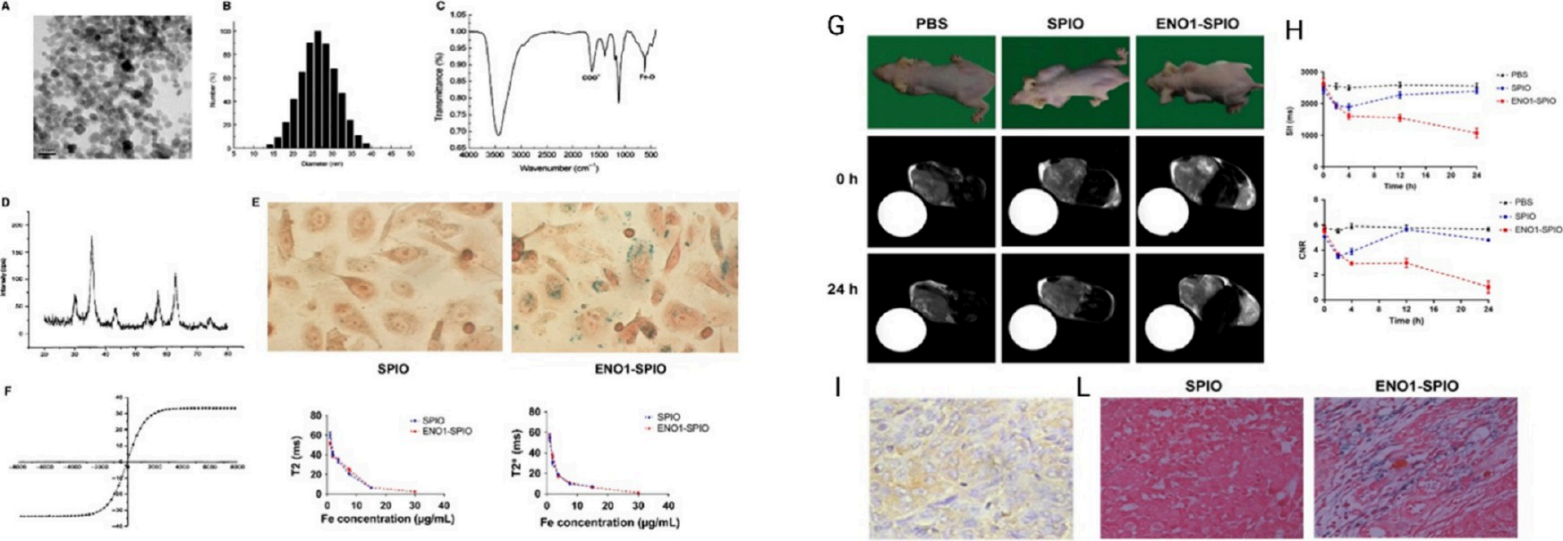
(Left panel) Characterization of ENO1-Dex-*g*-PCL/SPIO nanoparticles (A–F). (Right panel) Detection of pancreatic tumor by in vivo MRI of ENO1-Dex-*g*-PCL/SPIO nanoparticles. (A) MRI of ENO1-Dex-*g*-PCL/SPIO nanoparticles in a pancreatic cancer xenograft mode. (B) Compared with the PBS control group and SPIO group, the T2 signal intensity of tumor tissue significantly decreased, and the peak of enhancement was at 24 h in the ENO1-SPIO group. (C) IHC staining (40×) of the pancreatic tumor tissues 24 h after injection with ENO1-SPIO nanoparticles. (D) Prussian blue staining (40×) of the pancreatic tumor tissues 24 h after injection with ENO1-SPIO or SPIO. More positive iron particles were found in the ENO1-SPIO group (G, H, I, L). Reprinted or adapted with permission under a Creative Commons CC BY 4.0 license from ref 84. Copyright 2020 Wiley.^84^

Active targeting via surface-conjugated antibodies is widely used to improve NP specificity for tumor cell. Triple single chain antibodies (scAbs) have been assembled to detect and treat PDAC by targeting multiple antigens.^67^ Compared to traditional monoclonal antibodies, their smaller molecular weight allow them to have less hindrance and favor escape from reticuloendothelial system phagocytosis after entering the body.^87,88^ In human PDAC cell lines BxPC-3 and SW 1990, SPION-scAbs were found to have significant toxicity at concentrations >150 μg/mL of scAbs after 72 h. The scAbs allowed specific binding and internalization by PDAC cells expressing MUC4, CEACAM6, and CD44v6. This also allowed for a shorter MRI *T*_2_ signal intensity with a transverse relaxivity (*R*_2_) measured at 104 mM^–1^ s^–1^, indicating strong MR contrast enhancement capabilities.^67^ Strengthening these results, *in vivo* studies on female nude mice with subcutaneous PDAC tumors showed a similar *T*_2_ signal decrease in the tumor region, with intensities decreasing to 40% at 6 h and 66.8% at 24 h postinjection.^67^

The enhanced sensitivity and specificity of using polyclonal antibodies provide a more accurate detection in MRI compared to conventional agents, also offering an opportunity to inhibit tumor growth. Mahajan et al.^89^ developed a theranostic siRNA-functionalized SPION imaging probe to deliver and monitor drug bioavailability in syngeneic orthotopic and genetically engineered mouse models. Significant biodistribution of the formulated SPIONs in the tumor was observed by enhanced contrast and thus reduction in *T*_2_ values.^89^ In addition to MRI enhanced contrast, tumor progression was slowed, as quantified by a decrease in proliferation and increase in apoptotic nuclei. In general, SPIONs, alone and used as hybrid nanosystems, provide higher sensitivity and specificity for tumor imaging by binding to specific tumor epitopes. This not only ensures better tumor visualization compared to nonspecific conventional agents but also enables the delivery of therapeutic drugs.

##### Stromal-Degrading Liposomes

Liposomes are phospholipid bilayer spherical vesicles that can encapsulate hydrophilic drugs or hydrophobic compounds on its membrane.^90^ Currently, FDA-approved liposomal-based drugs, such as Doxil and Onivyde, are used to enhance the delivery of chemotherapeutic agents to the tumor and limit systemic exposure and associated toxicity, commonly seen with traditional free drug chemotherapy.^91,92^ Their ability to carry and deliver imaging probes such as gadolinium for MR contrast has also been tested. In a study involving orthotopic pancreatic tumor xenograft and patient-derived xenograft (PDX) mouse models, gadolinium-based liposomes demonstrated their biodistribution in tumor tissue. Only 1% of the injected dose reached the PDAC tissue.^71^ MRI analysis of the free Gd control group showed its clearance by the urinary system during the first 30 min postinjection. Meanwhile, the leftover liposomal Gd was found circulating in the blood accumulating in the tumor following 24 h, confirmed by inductively coupled plasma optical emission spectrometry (ICP-OES).^71^ In general, this finding emphasizes the struggle faced in NP uptake by PDAC biological barriers. Pretreatment with collagenase-based liposomes demonstrated to increase therapeutic agent penetration by proteolytic enzyme degradation of collagen, a major key component in the PDAC stroma.^93^ Similar biodistributions were obtained in other studies. Solid magnetoliposomes (SMLs), composed of a solid iron oxide core and a phospholipid layer, demonstrated a similar accumulation pattern, as illustrated in the previous study. Along with stroma-modulating inhibitor sonic hedgehog (sHH), SMLs increased their tumor permeability and deposition resulting in a tripling of *T*_2_ contrast after 24 h relative to sHH-untreated controls (2.0 ± 1.7 vs. 5.9 ± 2.4 s ^–1^)^72^ (Figure 13). This further confirms the therapeutic importance of stromal modulation upon tumor NP deposition.

**Figure 13:**
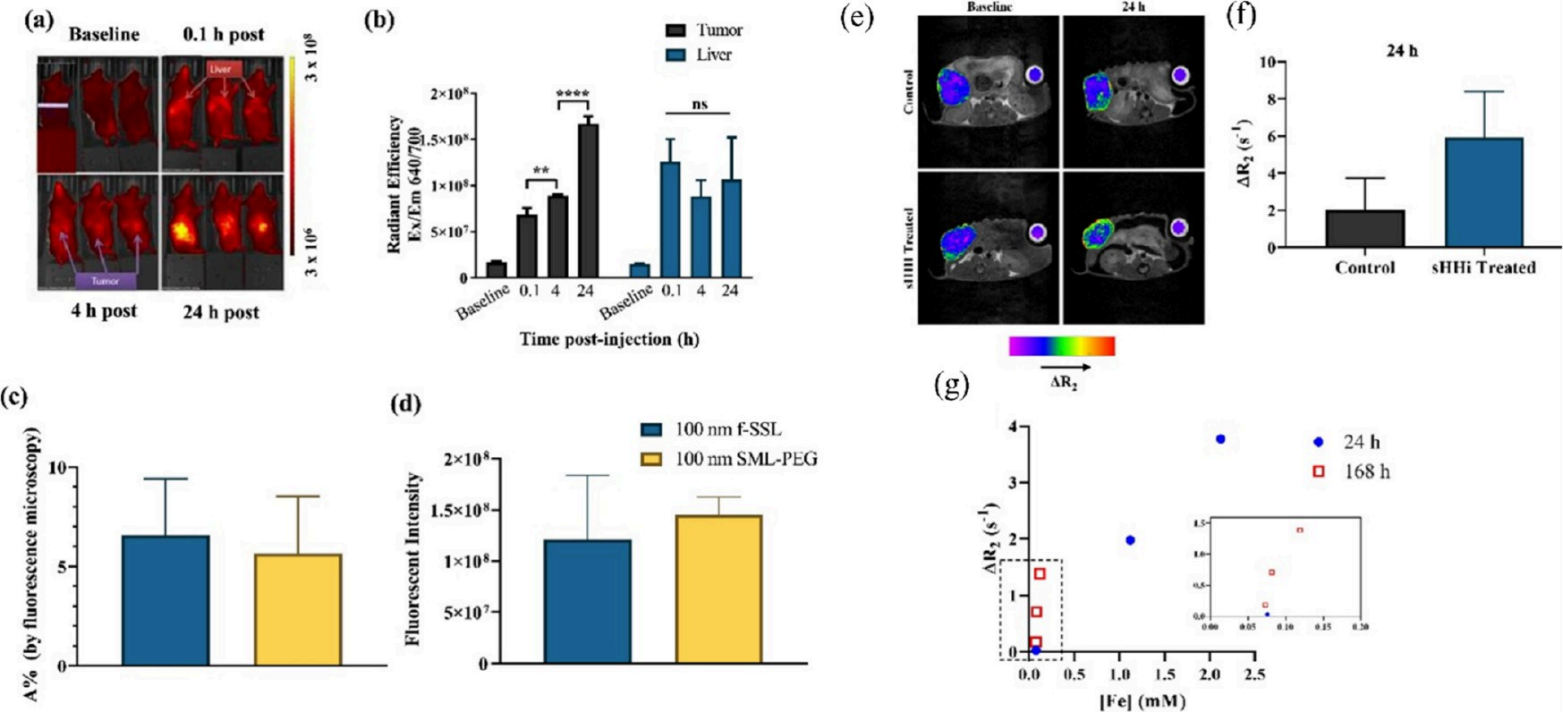
(a) IVIS images of 100 nm SML-PEG biodistribution in SCID mice bearing MIAPaCa2 PDAC tumors. Tumor deposition increased over time, whereas liver deposition peaked shortly after injection. (b) Quantification of SML-PEG in tumor and liver at 0, 4, and 24 h post injection, showing a continuously increasing deposition in the tumor. (c, d) Mice bearing PDAC PDX #18269 were injected iv with SML-PEG or fluorescent SSL. (e) Representative MRI slices of tumors overlaid with R 2 maps before and 24 h after SML-PEG injection (Fe dose 50 mg/kg) in control and primed (sHHi) mice. (f) Average recorded R 2 for #18269 tumors 24 h after SML-PEG injection in control and sHHi-primed mice, showing (g) a threefold increase following sHHi-priming. Reprinted or adapted with permission under a Creative Commons CC BY 4.0 license from ref 72. Copyright 2023 Elsevier.^72^

In general, NPs possess intrinsic and unique physiochemical properties that aid MRI capabilities for PDAC tumor diagnosis. Their multifunctionality and targeting capacity provide a substantial advantage when compared to traditional imaging agents, such as chelated gadolinium, which lack dual modality and undergo rapid systemic clearance, resulting in short circulating half-lives and limited long-term monitoring capabilities. The various natural and synthetic biomaterials used to create these NPs promise choice flexibility when deciding what type of therapy to use. Polymeric NPs are known for their versatility and biocompatibility and can be functionalized with targeting ligands, enhancing their efficacy as MRI contrast agents in PDAC diagnosis. Inorganic NPs, such as gold- and iron oxide-based ones, offer great tunability, leveraging their electrochemical and magnetic properties for superior diagnostic imaging applications. Liposome-based NPs have little, although enough for an MRI signal enhancement, tumor accumulation. The incorporation of stromal-degrading proteases can negatively modulate the dense, collagen-rich extracellular matrix (ECM) in PDAC tumors, improving the penetration and delivery of these NPs.

## 4 Challenges in the Tumor Microenvironment

As described, the development of NP-based MRI has the potential to improve the outcomes from conventional contrast agents; however, the true diagnostic impact of these nanosystems is dependent on their ability to penetrate, accumulate, and persist within PDAC tissue. These processes are profoundly influenced by the tumor microenvironment characteristics, including stromal density, desmoplasia, vascular collapse, and elevated interstitial fluid pressure, all of which NP design and restrict effective delivery and that will be discussed in this chapter. The high density of the stroma creates physical barriers that hamper NP penetration and distribution within the tumor. Additionally, the PDAC microenvironment is poorly vascularized leading to high interstitial fluid pressure (IFP), which collapses surrounding blood vessels and further hinders delivery.^94^ Therefore, targeting strategies that exploit the leaky blood vessels to passively accumulate in the tumor interstitium, such as the EPR effect, are limited in PDAC.^95^ To mitigate this, MacCuaig et al.^96^ studied size-dependent mechanisms of NP uptake in *in vivo* orthotopic mice. Reducing the size of mesoporous silica NPs, in a range between 30 and 50 nm, did indeed improve tumor penetration; however, the uptake efficiency was dismal when compared to the active targeting ones. Targeting the ECM’s collagen and cancer-associated fibroblasts (CAFs) is also another strategy to pave the way for NPs to infiltrate inside the tumor. Degrading collagen reduces stromal stiffness and density; as such, enzymes that break down extracellular structures, like collagenases, have been shown to decrease IFP and enhance vascular perfusion allowing improved NP penetration.^71,97^ CAFs are a dominant cell type in the PDAC stroma that directly contribute to the production of collagen family of proteins.^97^ Repurposing of drugs like aspirin have been studied for their antidesmoplasia effects that target CAFs. However, their encouraging efficacy was limited only at the preclinical stage.^98^ Recently, Nicolás-Boluda et al.^99^ successfully depleted the CAF population in a cholangiocarcinoma model by using AuNP-mediated photothermal therapy decreasing tumor’s stiffness and progression. The major disadvantage of degrading the ECM is the potential for metastasis formation since cancer cells are no longer tightly withheld in the TME that impede their escape.^97^ The transcytosis intracellular mechanism involves the uptake of NPs across the endothelial cell layer via vesicular transport. Functionalized NPs with specific ligands can bind to respective overexpressed receptors on endothelial cells, triggering endocytosis. Examples include albumin ligands that can bind to the gp60 receptor, and the urokinase plasminogen activator-receptor (uPAR) for targeted pancreatic cancer imaging and therapy.^95,100^ Similarly, micropinocytosis, a process by which cancer cells acquire nutrients from the extracellular environment, is highly promoted in PDAC cells. Albumin-functionalized NPs can act as a camouflaged nutrient source for PDAC cells in order to be internalized,^75^ and various tests have been performed in vitro about cellular uptake of functionalized nanoparticles.^76^ PLGA surface-treated nanoparticles with bovine serum albumin (BSA) and adenosine (ADN) showed a higher uptake in MiaPaca2 and Panc1 cell lines, confirming cells’ preference to endocytose albumin for glutamine production and adenosine.^76^ The increased specificity of ligand-mediated transcytosis enables a more precise delivery of NP-based imaging probes to PDAC cells, reducing off-target effects and potentially enhancing MR diagnostic capabilities. NP uptake in PDAC involves a combination of passive and active mechanisms. The major delivery obstacles are represented by the dense and fibrotic tumor environment. The EPR effect provides a basis for accumulation, with its efficacy limited by the tumor dense stroma. Transcytosis offers a more effective pathway for NP delivery, especially when combined with strategies to modify the ECM. Understanding these mechanisms is crucial for designing NPs that can effectively penetrate and accumulate in PDAC tumors, improving diagnostic and therapeutic outcomes. Future directions should focus on optimizing the NP design for better interaction with the PDAC and TME.^101,102^

This includes developing multifunctional NPs capable of simultaneous imaging and therapeutic delivery, enhancing their stability and circulation time and improving targeting efficiency through advanced surface modifications. Furthermore, clinical translation will require robust validation in preclinical trials, addressing many potential safety concerns related to long-term use and biodegradation. Figure 14 provides an overview of the stromal targets for these nanoformulations.

**Figure 14:**
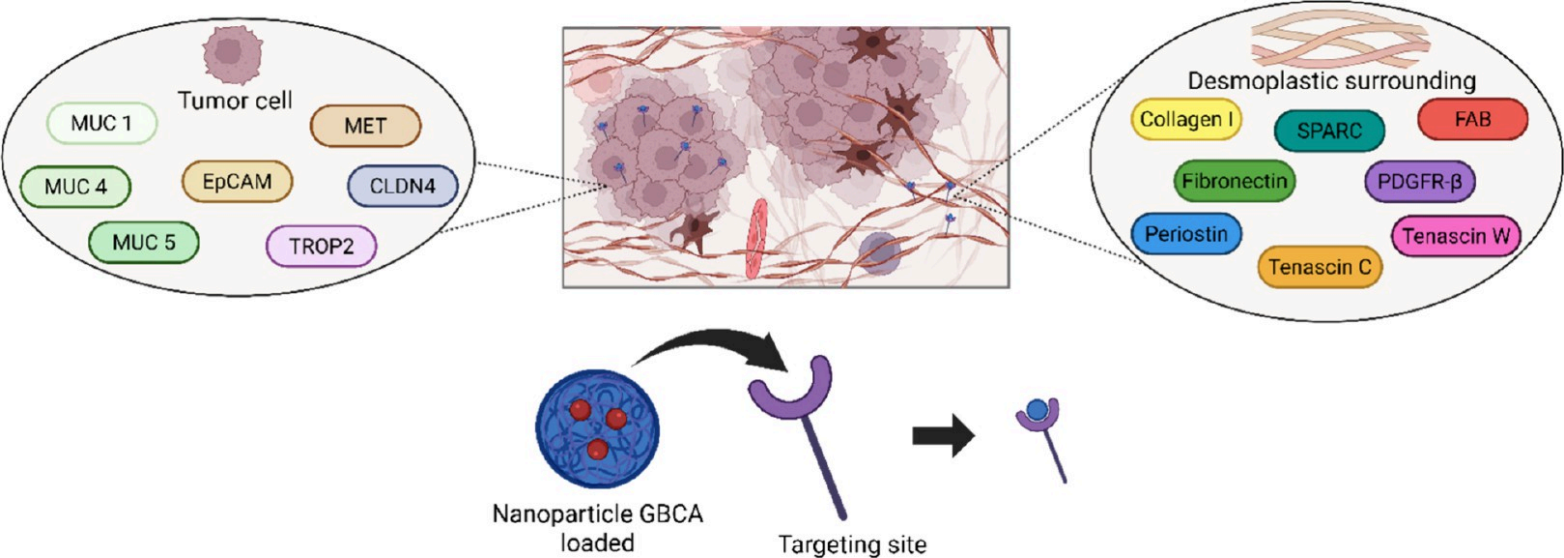
Cellular and desmoplastic sites of interest in targeted MRI.

## 5 Targetable Nanosystems for Early or Late Diagnosis of PanIN Lesions

Several challenges need to be addressed before clinical translation, primarily due to the complex and obstructive tumor microenvironment (TME). The dense, desmoplastic stroma characteristic of PDAC, which includes the collagen-rich extracellular matrix along with cancer-associated fibroblasts, hampers the effective delivery and distribution of contrast material. This microenvironment causes elevated interstitial fluid pressure, which reduces the perfusion and diffusion of nanoparticles within the tumor, impairing delivery of systemic therapies^103^ Additionally, stromal heterogeneity in PDAC creates variable biological barriers to particle penetration and retention. These barriers complicate the imaging capabilities by affecting their distribution and imaging contrast.^104^ The immunosuppressive nature of PDAC’s TME also limits the effectiveness of immune-targeted NP strategies, impacting the diagnostic potential of enhanced MRI contrast.^105^ Among the recognized precursors of PDAC, lesions such as pancreatic intraepithelial neoplasms (PAnINs), particularly PanIN high-grade ones, are the most frequent and have a high risk for malignancy progression. PanIN lesions occur in the small pancreatic duct, are less than 5 mm in extension, and cannot be detected directly with imaging techniques. The multistep progression reflects the accumulation of genetic mutations (*e.g*., KRAS, CDKN2A, TP53, and SMAD4) and histological changes that ultimately lead to invasive pancreatic cancer^106,107^ (Figure 15).

**Figure 15:**
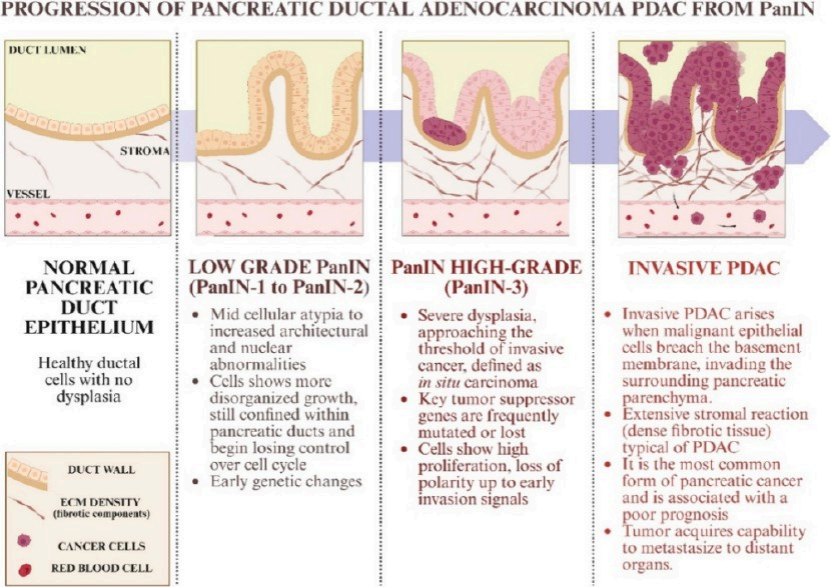
Steps of the tumor progression from normal pancreatic duct epithelium to invasive PDAC.

Considering the role of PanINs as a precursor to PDAC, and the limitations associated with its diagnosis, identifying targetable biomarkers that can both detect the disease at an early stage and assist in the surgical removal phase is of critical importance. PanIn diagnosis employs methods selected for each pathological marker—including histopathology, aberrant miRNA profiling, and metabolomic analysis—with imaging as the reference standard. Significant efforts are still underway to identify novel biomarkers that can distinguish between the various stages of disease progression. In addition to this, further research is still needed in understanding the potential of already known and not yet exploited markers. Vessels are neither fully compressed nor fibrotic, which facilitates probe delivery.^108^ A double approach can be considered for early-stage PDAC imaging: targeting of cellular and desmoplastic markers. Among cell surface markers, transmembrane mucins MUC1, MUC4, and MUC5AC are already known for their role as solid tumor biomarkers, in pancreatic, breast, lung, ovarian, and colorectal cancers, and are expressed from the earliest PDAC stages.^67,109–111^ Epithelial cell adhesion molecule (EpCAM) offers an alternative target, though it remains under-explored.^112^ Hepatocyte growth factor receptor (MET), Claudin-4 (CLDN4), and trophoblast cell surface antigen 2 (TROP2) exhibit low expression in PanIN-1 lesions but rise significantly by PanIN-2,3 stages, providing insight into tumor progression.^113,114^

Desmoplasia begins during the PanIN stages, particularly in PanIN-2,3, playing a critical role in the early remodeling of the tumor microenvironment (TME). Early markers of desmoplasia fibronectin and collagen I grabbed particular attention as they appear as fibrosis first sets in.^115^ Secreted protein acidic and rich in cysteine (SPARC), PDGFR-β, and fibroblast activation protein (FAP) are expressed at these stages and may guide NP delivery. Periostin and tenascin C peak later in tumor development. Tenascin W is recognized as a promising targetable marker, although limited literature is currently available.^116,117^ In-depth examination of these biomarkers could elucidate novel ways to aid MRI in detecting early lesions. Designing contrast agents that bind several targets at once may overcome low antigen levels and sharpen the resulting image.

## 6 Clinical Importance of NP-Based Imaging in PDAC

The translation of NP-based imaging agents into the clinic is of relevance for PDAC. Conventional MRI contrast agents are rapidly cleared and lack tumor specificity, reducing their effectiveness in detecting small lesions or monitoring the treatment response. NP formulations overcome some of these limitations by improving circulation half-life, enhancing local tumor accumulation, and enabling ligand-mediated specificity toward PDAC-associated biomarkers. From a clinical perspective, NP-based MRI offers several advantages. NPs can facilitate early detection by targeting precursor lesions such as PanINs or IPMNs, which are usually invisible to standard MRI and yet represent critical stages in disease progression. They also improve tumor delineation since their higher relaxivity and targeted accumulation allow radiologists to better distinguish PDAC from conditions like chronic pancreatitis or benign cystic lesions, which often mimic malignant features. Another important aspect is the possibility of real-time therapy monitoring: Theranostic nanosystems that combine imaging and drug delivery make it possible to track drug bioavailability and treatment efficacy directly within the tumor, which is not achievable with traditional contrast agents. Furthermore, the improved circulation profiles of NP carriers can reduce systemic clearance and allow lower doses of contrast material, potentially lowering the risk of adverse effects associated with free gadolinium or iron.^118^

Despite these advantages, several obstacles remain for clinical adoption. Heterogeneous stromal barriers, variability of the EPR effect among patients, and immune clearance mechanisms continue to limit the consistent tumor uptake. In addition, concerns about long-term biodistribution, large-scale reproducibility, and regulatory approval still need to be addressed.^119^ Nonetheless, the successful clinical use of liposomal irinotecan (Onivyde) in PDAC therapy and iron-based agents in other indications demonstrates that NP formulations can be translated into practice.^120^ These precedents highlight the strong potential of NP-based imaging as a future diagnostic and monitoring tool for PDAC.

### 6.1 Challenges for Clinical Translation of NP-Based Imaging

Preclinical studies highlight the potential of NP-based imaging for PDAC, although several barriers hinder their translation into clinical use. Regulatory approval requires extensive evaluation of safety, efficacy, and manufacturing reproducibility, yet most formulations remain at the experimental stage. A key challenge is large-scale synthesis: NPs that are reproducibly manufactured in small laboratory batches often lose uniformity, stability, or targeting efficiency when scaled up under Good Manufacturing Practice (GMP) conditions.^119^ This raises concerns about batch-to-batch variability, quality control, and long-term storage.

Another critical issue is the safety profile of the nanomaterials. While polymers such as PLGA are biodegradable, inorganic platforms like gold or silica may persist in tissues, raising questions about long-term accumulation and toxicity.^121^ Systematic toxicology studies, particularly for chronic use or repeated imaging sessions, are still lacking. In addition, the dense stromal environment in PDAC means that even safe and effective probes may show limited penetration in patients, reducing clinical benefit compared to preclinical models.^122^

Finally, the cost-effectiveness must be considered. Many of the advanced nanostructures involve complex multistep synthesis, functionalization with ligands or antibodies, and incorporation of expensive metals such as gold or gadolinium. These steps increase production costs, making widespread clinical use less feasible unless clear diagnostic or therapeutic advantages are demonstrated.^119^

Addressing these translational challenges will require not only optimization of NP design but also interdisciplinary efforts involving chemists, clinicians, regulatory agencies, and industry partners. Only through coordinated progress in safety validation, scalable manufacturing, and cost reduction can NP-based MRI agents undergo a transition from promising preclinical tools to practical options in PDAC diagnosis and monitoring.

Looking ahead, promising strategies to overcome these barriers include the design of biodegradable inorganic–organic hybrids to improve clearance, the use of stimuli-responsive coatings to enhance stromal penetration, and combination with stromal-modifying agents such as hyaluronidase or collagenase to improve uptake. Advances in microfluidics-based NP synthesis may address scale-up and reproducibility, while multimodal imaging approaches (e.g., MRI–PET and MRI–photoacoustic) could provide a stronger justification for clinical translation. Together, these approaches illustrate that despite existing limitations, NP-based MRI holds realistic potential to improve PDAC management if technical, biological, and regulatory hurdles are jointly addressed.

## 7 Conclusion and Perspectives: The Potential of NP-Based MRI in Shaping PDAC Diagnosis and Treatment

NP-based MRI could have high potential in shaping the future of early-stage PDAC diagnosis. The unique characteristics of the described NPs, such as their high surface-to-volume ratio and surface functionalization with targeting ligands, provide substantial advantages over traditional imaging methodologies, including enhanced MR contrast, enhanced biodistribution, and the capability for multimodal imaging, which collectively improve the sensitivity and specificity in PDAC detection. The integration of nanotechnology for MRI acquisitions has shown promising results in preclinical studies, with NPs demonstrating superior tumor penetration and imaging contrast compared to those of conventional agents. For example, superparamagnetic iron oxide nanoparticles (SPIONs) and AuNPs have been successfully used to enhance *T*_2_-weighted MRI signals and provide clear imaging of PDAC tumors.^123^ Multifunctional NPs that combine diagnostic and therapeutic capabilities, as theranostic agents, offer a dual benefit, allowing for simultaneous imaging and treatment of pancreatic associated tumors, which could significantly improve patient outcomes. However, a series of challenges for clinical translation are present and need to be addressed. One, above all, is the optimization of Np formulations to overcome the high-density PDAC stroma. Moreover, safety and biocompatibility issues are of great concern to establish standardized protocols for clinical use. In conclusion, NP-based MRI represents a promising frontier in the diagnosis and treatment of PDAC. With continued research and development, these advanced imaging agents have the potential to significantly improve early detection, providing more accurate diagnosis and enhancing therapeutic outcomes of patients, ultimately shaping the future of cancer diagnosis and treatment.
